# Implementation of a SenseMaker® research project among Syrian refugees in Lebanon

**DOI:** 10.1080/16549716.2017.1362792

**Published:** 2017-08-31

**Authors:** Nour Bakhache, Saja Michael, Sophie Roupetz, Stephanie Garbern, Harveen Bergquist, Colleen Davison, Susan Bartels

**Affiliations:** ^a^ Department of Public Health Sciences, Queen’s University, Kingston, ON, Canada; ^b^ Sexualities and Sexual and Reproductive Health and Rights, ABAAD Resource Center for Gender Equality, Beirut, Lebanon; ^c^ University of Leipzig, Department of Psychology, Leipzig, Germany; ^d^ Department of Emergency Medicine, Beth Israel Deaconess Milton, Milton, MA, USA; ^e^ Brigham and Women’s Hospital, Department of Emergency Medicine, Boston, MA, USA; ^f^ Department of Emergency Medicine, Harvard Medical School, Boston, MA, USA; ^g^ Department of Emergency Medicine, Queen’s University, Kingston, ON, Canada; ^h^ Kingston General Hospital Research Institute, Kingston, ON, Canada

**Keywords:** Child marriage, early marriage, SenseMaker®, Syria, tablet

## Abstract

The Syrian conflict has displaced over 1.2 million Syrians into Lebanon. As a result of displacement, some Syrian families are turning to child marriage as a coping mechanism. The prevalence of early marriage has reportedly increased and the average age of marriage decreased during the crisis. The aim of the project was to understand the underlying factors contributing to child marriage among Syrian refugees in Lebanon using Cognitive Edge’s SenseMaker®. This manuscript explores the process of implementing this novel research tool in a humanitarian setting. Twelve interviewers conducted SenseMaker® interviews with married and unmarried Syrian girls, Syrian parents, as well as married and unmarried men. Participants were asked to share a story about the lives of Syrian girls in Lebanon and to self-interpret the narratives by answering follow-up questions in relation to the story provided. Data collection occurred across three locations: Beirut, Beqaa, and Tripoli. In total 1422 narratives from 1346 unique participants were collected over 7 weeks. Data collection using SenseMaker® was efficient, capable of electronically capturing a large volume of quantitative and qualitative data. SenseMaker® limitations from a research perspective include lack of skip logic and inability to adjust font size on the iOS app. SenseMaker® was an efficient mixed methods data collection tool that was well received by participants in a refugee setting in Lebanon. The utility of SenseMaker® for research could be improved by adding skip logic and by being able to adjust font size on the iOS app.

## Background

Since 2011, the Syrian conflict has resulted in more than 250,000 deaths, two million people wounded, and 11 million people displaced [1]. The scope of the crisis has resulted in substantial social and economic challenges for Syrian refugees, including the 1.2 million Syrians [2] who have been displaced into Lebanon [3]. Many Syrian refugees in Lebanon are living in precarious circumstances in informal settlements with few legal rights [4]. In 2014, approximately 70% of the Syrian refugees registered with the United Nations High Commissioner for Refugees (UNHCR) in Lebanon were considered poor with increasing debt and a growing reliance on food aid [4]. Furthermore, half of the refugees are children and most face challenges with enrolling in educational institutions [5,6]. Over 250,000 school-aged Syrian children are currently out of school [5,6].

Child marriage is defined as ‘*any formal or informal union where one or both parties is below the age of 18*’ [7]. Child marriage is widely recognized as a human rights violation, as a form of gender-based violence (GBV), as a violation of the Convention on the Elimination of All Forms of Discrimination against Women [8], as well as a violation of the Convention on the Rights of the Child [9–12]. Although child marriage occurred in some areas within Syria prior to the war, with 13.3% of girls under the age of 18 reportedly married in 2006, forced displacement has resulted in a change in child marriage trends [13]. More specifically, the prevalence of married girls under 18 years of age has reportedly increased while the age at marriage has reportedly decreased over the past 5 years [14–17].

As a result of the difficulties associated with displacement, child marriage has been identified as a coping strategy among Syrian refugee households [5,18]. Financial hardship and continuous concerns about protecting girls from sexual violence lead some families to marry their daughters early as a way to secure their futures financially and to protect them from sexual violence [18]. Child brides are at high risk for pregnancy and labor complications including preterm labor, obstructed or prolonged labor, and obstetrical fistulas [10,11]. Infants born to young mothers are also at greater risk of low birth weight, neonatal death, and stillbirth [7,10,12,19]. Additionally, experiences of physical, psychological, and sexual violence are more prevalent among girls who marry as children than among those who enter into marriage as consenting adults [7,10,12].

Our ultimate aim is to reduce the rates of child marriage among Syrian refugees and to do so we used a novel research tool, Cognitive Edge’s SenseMaker®, to collect mixed methods data regarding the underlying factors that contribute to child marriage with the goal of identifying local interventions and community actions to address the issue. SenseMaker®, available as an app for smartphones/tablets and as a browser-based software program, allows the collection of large quantities of data, in a short period of time. Qualitative data is collected as brief narratives documented in the form of text or audio recordings. Using a tablet or smartphone, participants share a personal story in response to their choice of three open-ended prompting questions, and then self-interpret their story by answering a series of follow-up questions as they relate to the events in the story. SenseMaker® allows for the collection of hundreds to thousands of self-interpreted narratives, providing results that have statistical power while still being rich in context. After initial analysis is complete, SenseMaker® data can be presented back to community members through focus group discussions to gather participants’ interpretation of and reaction to the results. These facilitated focus group discussions can also serve as a medium to identify feasible and culturally appropriate interventions that address child marriage.

We choose SenseMaker® for this research project because it offered a mixed quantitative/qualitative approach with digital data entry that we believed would be more efficient. Additionally, given the sensitivities of discussing child marriage, we hypothesized that SenseMaker®’s open-ended questions might provide more revealing and honest responses than would be possible with more directed questioning. Furthermore, because the SenseMaker® self-interpretation questions (triads, dyads and stones) allow for more varied responses than typical categorical questions, we believed it might provide a more nuanced understanding of the complexities around child marriage. And finally, we were interested in reducing researcher bias which was possible with SenseMaker® since the qualitative data is self-interpreted by the participant.

Thus far, documentation of SenseMaker® use among refugee populations and in humanitarian settings is limited [20]. Therefore, the purpose of this manuscript is to address this gap by providing a scholarly reflection on the process of implementing this novel research tool in a humanitarian setting and examining the challenges and opportunities associated with doing so. SenseMaker® data analysis will not be reviewed here and the quantitative/qualitative research results will be presented separately.

## Methods

### Sampling

To capture varied perceptions of the life of Syrian girls, a wide range of participants were targeted including married and unmarried Syrian girls, Syrian mothers and fathers, the husbands of Syrian child brides, unmarried men who may choose to take a child bride, and community leaders such as teachers, health care providers, religious leaders, members of the NGO community, etc. The percentage of Syrian refugees in each of the three target locations was used to calculate a purposeful weighted sample size for each geographic area of focus. Although a formal sample size calculation is not appropriate for this mixed qualitative/quantitative data collection, Cognitive Edge recommends a minimum of 300 stories per location. This heuristic allows one to disaggregate the data based on subgroups while still maintaining at least 50 stories per subgroup. A minimum of 300 stories per site also provides enough data points to allow patterns to emerge on visual scanning of the triads, dyads and stones. In addition, we aimed to interview 45 community leaders across the three regions. A convenience sample was used and participants were recruited in a targeted fashion to meet thresholds in each participant subgroup per location. Table 1 shows the number of participants interviewed in each subgroup.

**Table 1.** Number of participants in each subgroup per location.

	Beirut	Beqaa Valley	Tripoli	Subgroup total	Overall total for group
**Married Syrian girls**	58	72	67	197	427
**Unmarried Syrian girls**	78	70	82	230	
**Syrian mothers**	83	88	74	245	443
**Syrian fathers**	63	83	52	198	
**Men married to Syrian girls**	70	66	92	228	496
**Unmarried men**	92	79	97	268	
**Community leaders**	23	13	20	56	56
**Total for location**	467	471	484	-	1422

### Survey development

**Figure 1.**
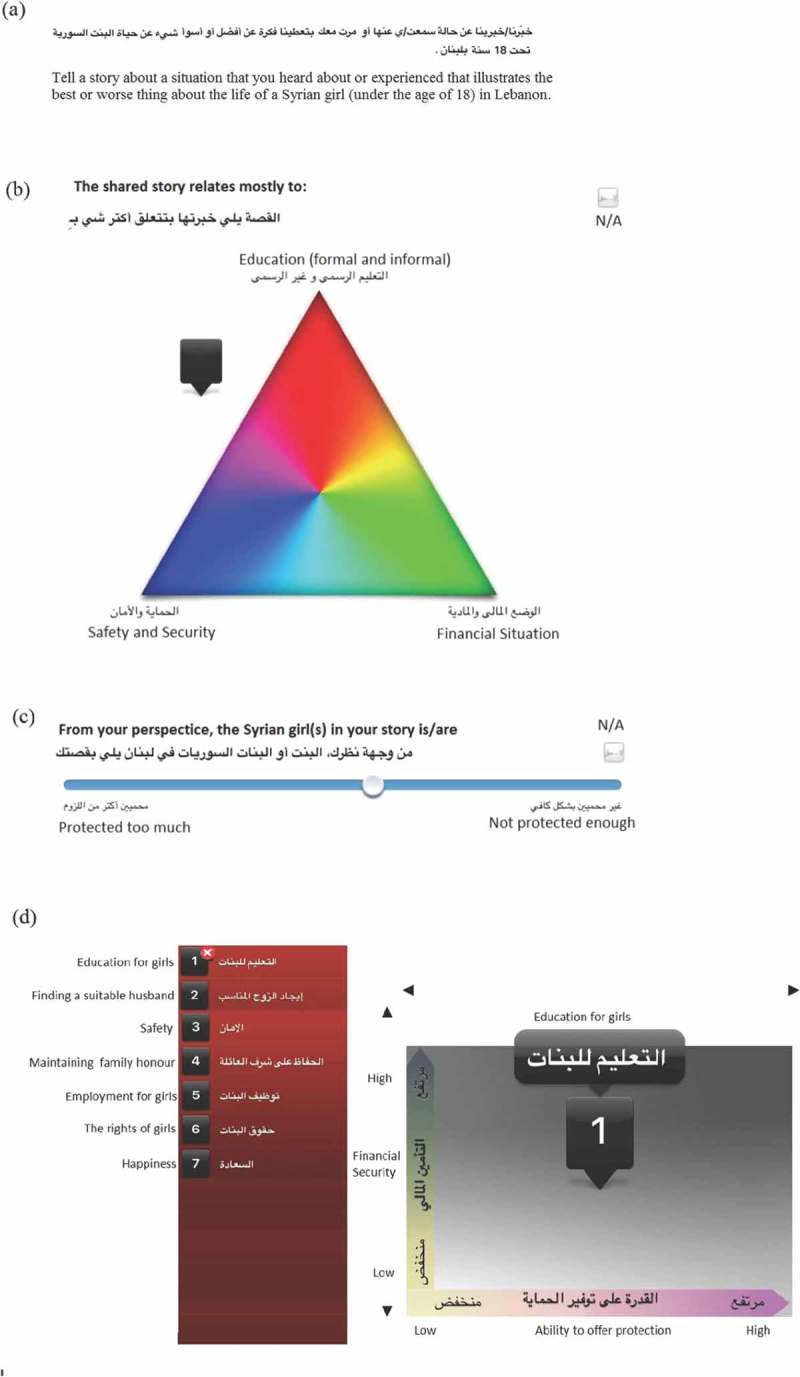
Categories of SenseMaker® questions. (a) Story prompts: Participants choose one of several prompts about which to share a narrative. (b) Triads: Participants move a marker between the three options in each triad to provide a more nuanced answer. (c) Dyads: Participants move a marker between two options using a slider. (d) Stones: Participants choose stones relevant to their shared story and plot them on the grid.

The SenseMaker® survey was drafted by team members with collective expertise on humanitarian crises due to armed conflict, child marriage, and survey design. Questions were reviewed iteratively by an experienced SenseMaker® consultant as well as by team members from the ABAAD Resource Center for Gender Equality in Lebanon and revisions were made accordingly. The survey began with three open-ended prompting questions, asking the participant to share an anonymous story about the experiences of Syrian girls in Lebanon (Figure 1(a)). After their stories were recorded, participants were asked to self-interpret their own narratives by responding to three different categories of SenseMaker® questions: triads (Figure 1(b)), dyads (Figure 1(c)), and stones (Figure 1(d)). The survey ended with a series of multiple-choice demographic questions in addition to questions used to contextualize the events shared in the story (e.g. emotional tone, the frequency of events). The survey was drafted in English, translated to Arabic by a Syrian translator, and then back translated to English to check for accuracy. Translation discrepancies were resolved by consensus. Once finalized, the Arabic survey was uploaded to the Cognitive Edge secure server and reviewed for errors prior to initiation of pilot data collection. The open-access SenseMaker® app was installed on each of the study’s tablets (iPad Mini 4) and the survey was downloaded into each app.

### Piloting

In May 2016, the SenseMaker® survey was piloted among 28 participants recruited through the ABAAD Resource Center for Gender Equality, across three target locations: Beirut (*N* = 10), Beqaa (*N* = 8), and Tripoli (*N* = 10). Two researchers (NB, CD, SM, or SB) were present at each interview, one to conduct the interview in Arabic, and the other to take notes on the interview process and to record any identified problems. At the end of the interview, participants were asked questions regarding the survey including opinion on length, comfort using the iPad, and difficulty responding to the different categories of SenseMaker® questions. The major issues identified in the pilot were difficulty with understanding the stones questions, discomfort with voice recording by some participants, and a tendency to answer the triads in the extreme margins. Survey revisions were made based on the pilot and the interviewers received dedicated instruction on how to deal with such issues if they occurred during the data collection.

### Training

Twelve interviewers were identified by the ABAAD Resource Center for Gender Equality based on their place of residence (Beirut, Tripoli, or Beqaa), gender, and nationality. There were four interviewers for each of the three locations with an equal number of males and females. To adequately capture the perspectives of both Lebanese and Syrian men, half of the six male interviewers were Syrian and half were Lebanese. Thus, each location’s interview team consisted of two Syrian females, one Syrian male, and one Lebanese male. All interviewers had completed secondary school, with the majority having completed or in the process of completing a university degree. Furthermore, a majority of the interviewers were already known to staff at the ABAAD Resource Center for Gender Equality through previous programs. Interviewer training was conducted during a four-day workshop in Beirut immediately prior to initiation of data collection in July 2016. The training was led by three of the study researchers (SG, SM and SB). Simultaneous translation with a professional Arabic-English interpreter was provided for all training activities. Day one of training provided an introduction to the study, an overview of SenseMaker®, a discussion around research ethics, and basic training on the use of an iPad. The second and third days consisted of practicing how to obtain informed consent, a detailed review of the SenseMaker® survey, multiple role-playing sessions, and training on the management of adverse events and program referrals. The last day of training consisted of reviewing data upload procedures and relevant troubleshooting, an overview of logistics including completing study ledgers, distribution of all study materials and a training debrief/feedback session.

All interviewers were trained on the GBV standard operating procedures for referral, as a mitigation measure for adverse events. Additionally, each participant was offered an ABAAD referral card that contained a hotline number for support and for information on how to access holistic GBV services. Additionally, interviewers were well informed to bring any adverse events to the immediate attention of their team lead. No adverse events were reported during the study period.

At the end of training, each interviewer received an iPad loaded with the SenseMaker® app and our child marriage survey, print copies of the training materials, print copies of data collection ledgers, referral cards and contact lists of study team members.

Three ABAAD field program staff members were assigned as ‘team leads’ for each of the geographic regions to serve as local points-of-contact for assistance with identifying study participants, facilitating logistics, troubleshooting technological problems and ensuring that data collection progressed as planned. Each of these team leaders reported to an ABAAD project manager and received brief individual training from the research team prior to data collection.

### Data collection

Data collection was conducted over a period of 7 weeks with close oversight by the team leads in each location in addition to the ABAAD project manager and two graduate students based in Beirut. Interviews were primarily conducted in the participants’ homes, at markets, at cafes, at support groups, or other points of contact with service providers. Shared stories were audio-recorded on iPads and participants then responded to a series of questions that provided quantitative data on the narrators’ interpretation of the experiences shared in the narrative. The project manager handled logistics, held regular phone meetings with each team lead and was immediately available via cell phone and *WhatsApp* to all interviewers ensuring that any identified issues were promptly addressed.

Several quality measures were regularly tracked during data collection. For example, location of the interview was to be recorded through GPS to ensure that participants were being sampled from diverse geographic locations. The length of each interview was also recorded with any interviews less than 10 minutes being flagged for review and the associated audio files checked against the participants’ responses. Furthermore, each interviewer was asked to report weekly on the number of unique participants interviewed in each subgroup using an online survey. Self-reported results were then compared to weekly data summaries from the Cognitive Edge server. In addition, the database was reviewed weekly to ensure there were no duplicate recordings.

The study protocol was reviewed and approved by the ABAAD Resource Centre for Gender Equality. ABAAD team members also provided extensive input when planning study implementation including logistics and security considerations.

## Results

### Facilitation of research

#### Efficiency

SenseMaker® proved to be an efficient data collection tool with 12 interviewers collecting 1422 self-interpreted stories (from 1346 unique individuals) over a 7 week period. SenseMaker® has several unique features that contributed to this efficiency: (a) typically the stories are relatively brief anecdotes and therefore they can be collected quickly, particularly if they are audio recorded; (b) the triads, dyads, stones and multiple choice questions allow the narrator to interpret their own story and through this self-interpretation, SenseMaker® generates quantitative data that is available almost immediately after upload to the server; (c) because the narratives have been interpreted by the participant to promptly generate quantitative data, it is not necessary to transcribe and translate all the stories thereby increasing efficiency and decreasing costs; and (d) SenseMaker® facilitated close monitoring and prompt feedback to interviewers regarding number of participants interviewed in each subgroup per location, quality of data, number of stories collected by each interviewer, etc. Based on our prior research experience, it would have been very difficult, if not impossible, to collect mixed-methods data on a sensitive topic from 1346 people across three locations in a 7-week period.

#### Mixed methods research

SenseMaker® provided rich mixed methods results since the accompanying narratives help to contextualize the quantitative data generated by the self-interpretation questions (triads, dyads and stones [Figure 1]). Using a large number of quantitative data points allows harvesting the ‘wisdom of the crowds’ and facilitates pattern recognition. The data is also connected to demographic information and so differences in subgroups can be further explored with substantial sample sizes. The qualitative stories are available to complement and provide depth to the quantitative data. This mixed methods approach allows for triangulation of qualitative and quantitative data across various participant subgroups and provides both breadth and depth of exploration.

#### Sensitive topics

Some of the shared Syrian girls’ experiences were of a sensitive nature. Direct questions regarding a delicate or polarizing issue such as child marriage could lead participants to answer in a way they feel the interviewer would agree with, thus introducing social desirability bias and potentially limiting the accuracy of the data. In this SenseMaker® survey, child marriage was not specifically introduced as a topic and there were no direct questions on child marriage. Despite this lack of direct questioning on the issue, 23% of the shared stories were recorded as being about child marriage (331 of 1423) and another 17% of the shared stories were recorded as mentioning child marriage (247 of 1423).

#### Costs

Implementing a SenseMaker® research project has unique associated costs, such as obtaining a license to use the software as well as fees for the upload and storage of data on the secure Cognitive Edge server. In this study, approximately 10% of the budget was dedicated to the use of SenseMaker® although this includes full analysis costs (approximately 5% for license, set-up and hosting and 5% for analysis). In addition to direct SenseMaker® costs, 12 iPads were purchased for data collection. Although there were less expensive tablets available, the SenseMaker® app functions most reliably on an iOS platform, leading us to invest in more these expensive devices. The iPads will remain the property of the study team for use in future research.

Despite some SenseMaker®-specific expenses, we believe that SenseMaker® was more cost efficient overall. The collection of 1422 self-interpreted stories using traditional research approaches would have taken longer thus requiring additional research assistant days as well as additional travel-related costs. Furthermore, the costs of uploading and storing data on the Cognitive Edge server were less than the costs that would have been required to have a research assistant digitally enter all the data. Direct digital entry also offered the advantage of having the data promptly available for analysis and eliminated data entry errors.

### Technology

#### Tablets

SenseMaker® is a tablet-based data collection tool and so reliance on technology was extensive in this study. Despite working in a humanitarian setting, this use of technology presented very few challenges. Being highly educated and comfortable with everyday use of technology, the interviewers were comfortable with using, charging and caring for the iPads as well as with accessing the Internet to upload data. We used the iOS SenseMaker® app in this study although Android and browser-based versions are also available.

#### GPS

Location tracking was inconsistent in the current study with GPS coordinates available for only 27% of interviews. Because the iPads used did not have cellular capability, they lacked internal GPS chips. Since Bluetooth and Wi-Fi were typically turned off to preserve battery power while interviewing in the field, without the internal GPS chip, GPS satellite coordinates could not be generated for a majority of the interviews. To improve on GPS tracking in the future, we would recommend keeping Bluetooth and/or Wi-Fi turned on to the extent possible given considerations of battery life. Alternatively, an external GPS tracking device could be plugged into the iPads, which would facilitate GPS tracking even in the absence of an internal GPS chip, Bluetooth capability or Wi-Fi access.

#### WhatsApp

During data collection, questions, concerns and updates were regularly communicated via the mobile phone messaging app ‘*WhatsApp’* [21]. Interviewers, team leads, and the project manager expressed a preference for *WhatsApp* as a means of communication, and so groups were created for the larger team of 12 as well as for each regional team. The ABAAD project manager and team leads often sent voice messages via *WhatsApp*. Correspondence via *WhatsApp* was an important asset for maintaining good team communication, building cohesiveness among the group, ensuring timely reporting and providing support to various levels of the research team when issues arose.

#### Voice recording

The audio quality of recorded narratives was good to excellent regardless of location and background noise. If the participant demonstrated discomfort with recording the story in his/her own voice, the interviewer listened to the participants’ story in full and the recounted it in his/her own voice for the purpose of recording (clearly stating at the beginning that the research assistant was recording the story on behalf of the participant). It is not possible to know how often the interviewer recorded the story on behalf of the participant without listening to all 1422 stories, which has not been done to date.

#### SenseMaker® programming

The SenseMaker® application itself had a number of programming limitations. First, the survey font size could not be altered, resulting in occasional instances of very small text that proved difficult for some participants to read. Second, use of Arabic punctuation caused some unanticipated challenges when data was extracted from SenseMaker®. For instance, Arabic commas were interpreted as delimiters in the extraction process, causing the exported text to shift and making it incomprehensible. This required the interview team to audio record the stories and to avoid the use of Arabic punctuation when they were typing responses. And third, SenseMaker® cannot currently perform skip logic, which results in some participants having to answer questions unnecessarily by choosing a ‘non-applicable’ option.

### Monitoring

At the end of each week, summary statistics from the survey were downloaded from the Cognitive Edge server and presented in Tableau, an interactive data visualization software program [22]. Each weekly summary included the cumulative number of stories collected from each of the participant groups in each of the three geographic locations, the cumulative number of interviews conducted by each interviewer, the cumulative number of stories about child marriage, length of each recorded interview and a link to allow quality control checks on the audio files. Review of the summary also allowed for easy identification of duplicates in the interviews, which sometimes occurred if the Internet signal was weak resulting in more than one attempt to upload the data. The final report on number of participants per subgroup and by location is provided in Table 1.

In addition to the monitoring components already mentioned, team leads were also responsible for providing reminders and following up with their interviewers about whether process measures were being followed on a daily basis and whether interviews were being conducted as per the training protocol. Team leads also held regional meetings approximately every 2 weeks, were responsible for overseeing sampling to ensure that participants were included from geographically diverse areas, and ensured that the iPads received maintenance and care including charging.

### SenseMaker® survey

The SenseMaker® survey was translated to the Syrian dialect of Arabic using language that was easy to read and understand, to account for varying levels of familiarity with formal Arabic particularly among the adolescent girls who had sometimes not completed primary school. Of the three types of SenseMaker® questions, the stones (Figure 1(c)) proved to be the most difficult for participants. For example, many participants would use all the stones regardless of whether the stone was relevant to their story. Furthermore, participants often plotted the stone on only one axis (either the X-axis or the Y-axis) and ignored the other axis. These challenges were recognized in the pilot and interviewers were therefore trained to use a three-step approach in guiding participants through the stone questions. Step one involved deciding whether a given stone was relevant to the story shared. Step two involved asking the participant to plot the stone along the X-axis and step three required the participant to then plot the stone according to the Y-axis, determining its final coordinates. This stepwise approach facilitated understanding of this complex style of question.

Another common issue was the participants’ tendency to choose extreme responses when answering the triads, dyads, and stones. Instead of the typical categorical responses that are most common in traditional quantitative surveys, SenseMaker® is designed to provide a more nuanced answer, and a tendency among participants to choose extremes limits that capability. Proneness to extremes was identified during piloting, and so interviewers were trained to explain to participants that having the marker between the different options is possible, and would allow them to give more complex answers.

In total, 59% of participants (840 of 1422) choose a single story prompt with distribution between the other two story prompts being more even (24% versus 17%). Although it appears that a single prompt resonated with a majority of participants, it is important to provide alternatives to increase the likelihood that at least one prompt will trigger memory of a relevant story for all participants.

## Discussion

We found SenseMaker® to be an efficient method of collecting mixed quantitative/qualitative data, facilitating the capture of 1422 self-interpreted narratives from 1346 unique participants by 12 interviewers over a 7 week period in Lebanon. Data was collected across three locations in a humanitarian setting.

Several SenseMaker® attributes contribute to its efficiency. First, rather than long, comprehensive transcripts as would be collected in more traditional qualitative research, the narratives captured by SenseMaker® are usually brief anecdotes, which in this case were audio recorded. This allows the interviews to be conducted more quickly with each lasting approximately 25–30 minutes. Second, through the use of SenseMaker®-specific questions (triads, dyads, and stones) as well as multiple choice questions, participants interpret their own narratives on the tablet, which the SenseMaker® app then uses to automatically generate quantitative data that is available almost immediately after uploading to the Cognitive Edge server. This eliminates the need to transcribe/translate transcripts and to code the data for identification of patterns and emerging themes, which is often very labor- and time-intensive. It also offers the additional advantage of reducing researcher biases that are inherent when investigators interpret the narratives of others. Based on data patterns identified in the triads, dyads, and stones, a selection of narratives can be transcribed/translated to contextualize the quantitative data but this is typically a relatively small subset of stories and allows the research team to save transcription/translation costs in addition to saving time. A final attribute contributing to SenseMaker®’s efficiency is that it facilitated close monitoring and prompt feedback by collecting the interviewer identification number, the participant subgroup, as well as the date and location of the interview. Because the data is captured digitally and is available promptly after upload to the Cognitive Edge server, this feedback can be provided to the research team in a timely fashion – daily if desired, although we found weekly to be sufficient for the purposes of this study.

Although it proved efficient, the SenseMaker® app has a number of limitations that might hinder its more widespread use for research purposes. For instance, within SenseMaker® the fixed font size was sometimes too small to be read comfortably on an iPad and it could not be enlarged. To ensure that font size does not compromise legibility for participants and interviewers, it is recommended that SenseMaker® be adapted to allow a variety of font sizes to meet individual needs. Additionally, SenseMaker® did not adapt well to Arabic’s non-Roman characters, which caused some text to shift during data extraction and rendered it nonsensical. Fortunately, the issue was recognized by the second day of data collection and was overcome by asking the interviewers to audio record the stories and to avoid the use of Arabic punctuation when they were typing responses. Although SenseMaker®’s limitations in working with Arabic were largely overcome in this study, we recommend that data extraction process be better adapted for use in different languages. Otherwise, its global usability will be limited. SenseMaker® also does not currently have skip logic capability, which results in some participants having to answer questions unnecessarily by choosing a ‘non-applicable’ option. This is inconvenient and lengthens the survey. Given the availability of numerous, less sophisticated survey programs that incorporate skip logic, this is a notable shortcoming of SenseMaker® and needs to be addressed if the application is to be taken up more broadly as a research tool.

Another disadvantage of SenseMaker® is that the narratives are briefer and sometimes lack the richness that might be found with more in-depth qualitative interviews. This is largely because the interviewers do not ask prompting or follow up questions. The impact of this is countered by having participants interpret their stories since they respond to the SenseMaker® questions with the full story in mind, adding additional layers of meaning even though they may not have articulated all the details in the audio recording. By collecting a large number of stories from a variety of stakeholders, SenseMaker® allows researchers to easily disaggregate the data to appreciate a variety of perspectives and this is certainly of value. In future research, however, we may consider combining SenseMaker® and its broad capture from community members with more in-depth qualitative interviews being conducted at a later time with participants who talk about particular topics of interest (in this case, child marriage).

## Conclusion

In summary, SenseMaker® offered several unique features that make it an efficient data collection tool to capture a large amount of mixed quantitative/qualitative data in a relatively short time period. It was feasible to use SenseMaker® in a humanitarian setting and the application appeared well suited to collect revealing and open perspectives on a sensitive topic. SenseMaker® also facilitated monitoring capability to easily track progress and to maintain quality assurance. However, the qualitative data collected with SenseMaker® tended to be short and less rich than would be expected with more traditional qualitative interviews and a number of programing limitations ought to be addressed to increase SenseMaker®’s utility for research purposes including ability to alter font size, to be more facile in other languages and incorporation of skip logic. In conclusion, although there is a capacity for improvement, SenseMaker® was found to be an efficient mixed-method data collection tool in humanitarian settings and among refugee populations.

## Paper context

SenseMaker® is a mixed method data collection tool available as an app for smartphones and tablets or as a browser-based software program. SenseMaker® use in humanitarian settings or among refugee populations is not well-documented. This study examines the challenges and opportunities of using SenseMaker as a data collection tool among Syrian refugees in Lebanon. Through these reflections, recommendations for future researchers and the improvement of SenseMaker® are provided.
